# Supplementary material for: Boron nitride nanoresonators for phonon-enhanced molecular vibrational spectroscopy at the strong coupling limit
Source: Light Sci Appl. 2018 Apr 6;7:17172–. doi: 10.1038/lsa.2017.172 (PMC6060053; doi:10.1038/lsa.2017.172)
Supplement: Supplementary Information [file lsa2017172x1.pdf]

Supplementary Information

Boron nitride nanoresonators for phonon-enhanced molecular vibrational spectroscopy at the strong coupling limit

Marta Autore[1], Peining Li[1], Irene Dolado[1], Francisco J. Alfaro-Mozaz[1], Ruben Esteban[2,3], Ainhoa Atxabal[1], Fèlix Casanova[1,3], Luis E. Hueso[1,3], Pablo Alonso-González[4], Javier Aizpurua[2,5], Alexey Y. Nikitin[2,3], Saül Vélez[1,**] and Rainer Hillenbrand[3,6,*]

[1] CIC nanoGUNE, 20018 Donostia-San Sebastián, Spain
[2] Donostia International Physics Center (DIPC), 20018 Donostia-San Sebastián, Spain
[3] IKERBASQUE, Basque Foundation for Science, 48013 Bilbao, Spain
[4] Departamento de Física, Universidad de Oviedo, 33007 Oviedo, Spain
[5] Centro de Física de Materiales (MPC, CSIC-UPV/EHU), 20018 Donostia-San Sebastián, Spain
[6] CIC nanoGUNE and UPV/EHU, 20018 Donostia-San Sebastián, Spain
[**] Present address: Department of Materials, ETH Zürich, 8093 Zürich, Switzerland.

[*]Corresponding author

S1 Extraction of CBP dielectric function

We measured the relative transmission spectrum of a 100 nm thick 4,4′-bis(N-carbazolyl)-1,1′-biphenyl (CBP) layer evaporated on top of a CaF_2 substrate. In order to extract the dielectric properties of the CBP molecules, we used the so-called Tinkham formula for thin films on a substrate[1]:

$$\frac{T}{T_0} = \frac{1}{\left|1+\sigma(\omega)d\frac{Z_0}{n+1}\right|^2} \tag{S1}$$

where d is the film thickness (d = 100 nm << $\lambda \approx 7$ μm), n is the refractive index of the substrate (n = 1.37 for CaF_2 in the considered range), Z_0 is the impedance of free space (377 Ω) and $\sigma(\omega)$ is the complex conductivity of the thin film. $\sigma(\omega)$ is related to the permittivity, according to $\varepsilon(\omega) = 1 + \frac{i}{\omega\varepsilon_0}\,\sigma(\omega)$ (ref. 2).

We model the permittivity of CBP in the range of interest as the sum of a dielectric non-dispersive background plus three Lorentz oscillators to describe molecular vibrations, as follows:

$$\varepsilon(\omega) = \varepsilon_\infty + \sum_k \frac{S_k^2}{\omega_k^2-\omega^2-i\omega\gamma_k} \tag{S1.2}$$

where S_k , ω_k^2 and γ_k represent the intensity, central frequency and damping of the k-oscillator. Fit is shown in Figure S1 (top panel, red dotted line). The two main molecular vibrations in the considered range, centered at ω_1 = 1450 cm^{-1} (γ_1 = 8.3 cm^{-1}, S_1 = 158 cm^{-1}) and ω_2 = 1504 cm^{-1} (γ_2 = 13.4 cm^{-1}, S_2 = 164 cm^{-1}) can be assigned to the in-plane deformation of the C-H bond mainly located on the carbazole rings and to the stretching of the C-N bond (combined to the in-plane deformation of the C-H bond mainly located on the biphenyl groups), respectively[3]. A third weaker vibration is characterized by ω_3 = 1478 cm^{-1}, γ_3 = 6 cm^{-1}, S_1 = 59 cm^{-1}.

With the parameters extracted from the fit, we are able to calculate the dielectric permittivity of CBP, according to Equation S1.2. The obtained $\varepsilon(\omega)$ is plotted in Figure S1, bottom panel. To verify the thin-film approximation, we used the fitting result $\varepsilon(\omega)$ to calculate the transmission spectrum (green curve in Figure S1), using the exact Fresnel coefficients. We find an excellent quantitative agreement with the measured transmission spectrum (black curve in Figure S1), which corroborates the validity of the thin-film approximation. By subtracting the green curve from the red curve, we find a maximum error of less than 0.5%

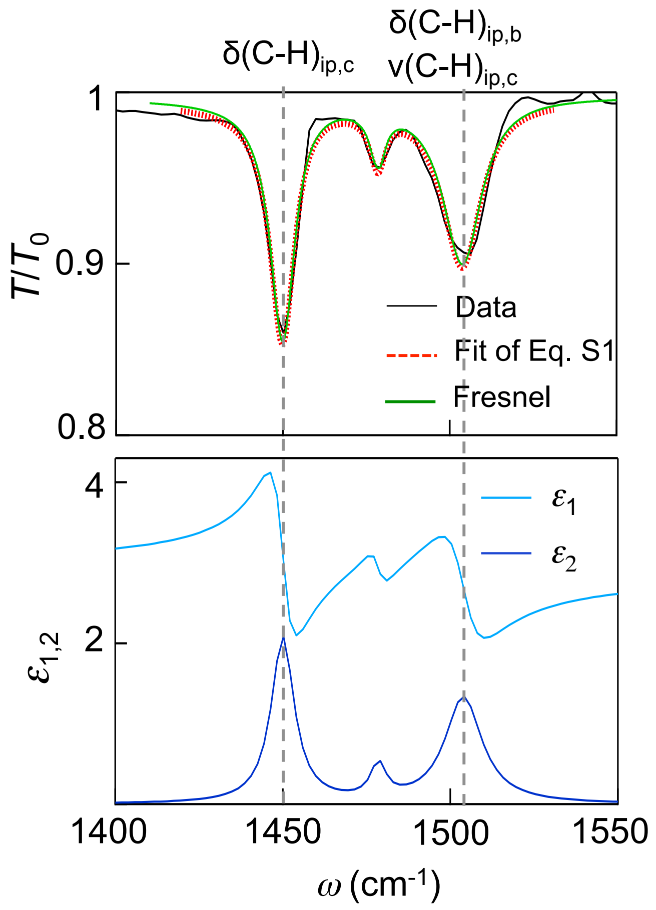

Figure S1. Extraction of the dielectric properties of CBP molecules. Upper panel: Relative transmission data of 100 nm thick film of CBP on top of a CaF_2 substrate (black). Fit to data of Equation S1 (red dashed). Exact calculation of CBP transmission based on Fresnel coefficients, using the dielectric function extracted by the thin film model (green). Lower panel: real (ε_1) and imaginary (ε_2) part of the dielectric permittivity, as calculated with the parameters extracted from the fit of transmission.

S2 Scanning electron microscope (SEM) image of h-BN ribbons

A SEM image of an h-BN ribbon array sample is shown in Figure S2. h-BN ribbons are colored light blue. Note that several surface imperfections and a certain rugosity are visible in the image, which could lead to increased scattering of phonon-polaritons and larger damping of Fabry-Pérot resonances. Moreover, the image reveals the trapezoidal section of the structures and the small width variations occurring along the ribbons, which may lead to inhomogeneous broadening of the HPhP Fabry-Pérot resonances.

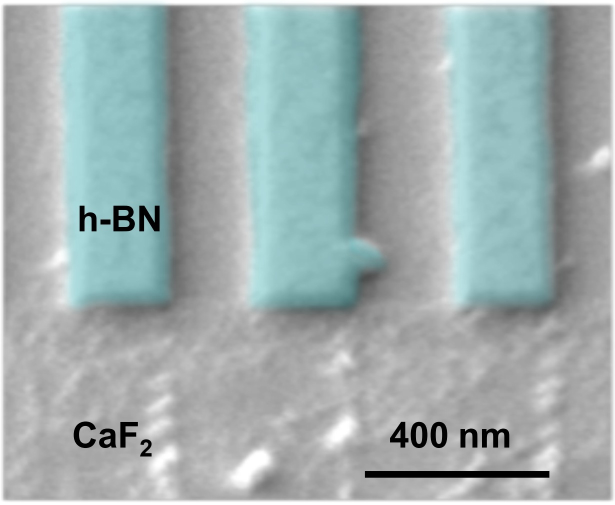

Figure S2. SEM image of h-BN ribbons on CaF_2.

S3 Extraction of central frequencies of transmission dips via Lorentzian fits

In order to extract the central frequencies of the dip-like features in transmission spectra of CBP covered h-BN ribbons (30 nm thick layer), which are associated to the coupled state of CBP vibration and HPhP resonance, we fitted single Lorentzian lineshapes to each dip.

The fits are shown in Figure S3 as red dotted lines, together with the data (black) for each measured sample. The central frequency of each dip is shown in Figure 4d of the main text (green dots).

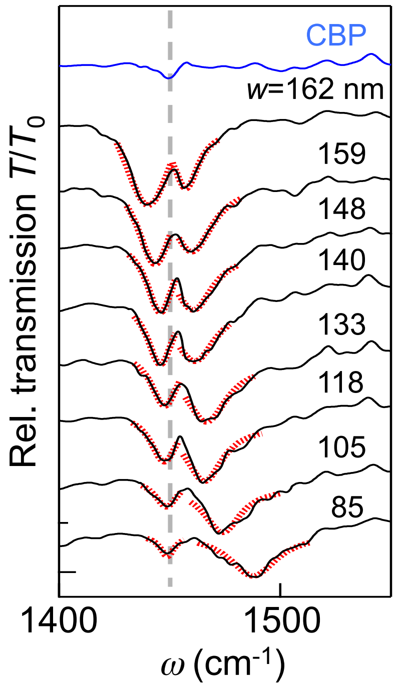

Figure S3. Lorentzian fits of transmission dips. Relative transmission of h-BN ribbon arrays with several widths, *w*, and fixed period *D* = 400 nm (black). Fit of single Lorentzians to each transmission dip (red dotted), in order to extract minima's positions.

S4 Coupled harmonic oscillators analysis for the CBP−h-BN ribbon system

In order to analyze the transmission spectra of the coupled CBP−h-BN ribbon arrays system, we describe it in terms of a classical harmonic coupled oscillators model, starting from the motion equations reported in the Materials and Methods section of the main text. Fits were performed according to $\frac{T}{T_0} = 1 - \mathcal{E} - Offset$, where $\mathcal{E}$ is the extinction, calculated as reported in the main text, and *Offset* is a constant. Moreover, the CBP central frequency and width have been fixed to the values extracted from the fit of the homogeneous 100 nm thick layer (see Figure S1), allowing for small variations, i.e. $\omega_{\mathrm{CBP}} = 1450.0 \pm 2$ cm^{-1} and $\gamma_{\mathrm{CBP}} = 8.3 \pm 1.5$ cm^{-1}. All fit parameters are reported in Table S1.

Sample	ω_{HPhP}	γ_{HPhP}	F_{HPhP}	ω_{CBP}	γ_{CBP}	F_{CBP}	*Offset*	g
**A1**	1446	24.2	3.3	1452	9.8	-0.08	0	7
**A2**	1454	24	3.1	1450	9.8	0.37	0	7.3
**A3**	1458.5	25.9	3	1450	8.3	0.42	0.02	6.3
**B5**	1458.2	23	2.9	1450	8.3	0.42	0	6.3
**A4**	1464.4	27.2	2.8	1451.1	8.3	0.24	0	6.4
**B6**	1465	25.3	2.9	1450.9	8.3	0.34	0	6.3
**A5**	1472.8	34.1	3	1452	8.3	0.18	-0.03	8.1
**B1**	1483.9	35.6	2.8	1451.4	9.8	0.43	0	8.1

Table S1. Parameters of the coupled oscillators model. All parameters are expressed in cm^{-1}, except for *Offset* (adimensional) and $F_{\mathrm{HPhP,CBP}}$ which are expressed in cm^{-2}.

We obtain the average values $\bar{g} = 7.0$ cm^{-1}, $\bar{\gamma}_{\mathrm{HPhP}} = 27.4$ cm^{-1} and $\bar{\gamma}_{\mathrm{CBP}} = 8.7$ cm^{-1}, which yield to C_1 = 0.37 and C_2 = 0.19, reported in the main text.

In Figure S4 we show a plot that summarizes the results of the coupled oscillators model fit to the transmission of the CBP−h-BN ribbon system. In particular, we plot the bare HPhP frequency ω_{HPhP} (black triangles), i.e. the frequency of the HPhP with no interaction, the bare CBP frequency ω_{CBP} (red circles), and the resulting eigenfrequencies $\omega_{\pm}$ (blue circles), as a function of the bare HPhP frequency ω_{HPhP}. In the same graph we plot the experimental minima of transmission, extracted via Lorentz fit (yellow squares).

From this plot, the avoided crossing behavior in the coupled system is clearly visible. Interestingly, the plot also unveils another important aspect of the coupled systems' analysis: the experimental separation between upper and lower branch of a coupled system in extinction measurements appears larger than the actual separation of the hybrid system eigenmode frequencies. This is an effect of the losses of the interacting modes and it has to be taken into account when analyzing the coupling regime.

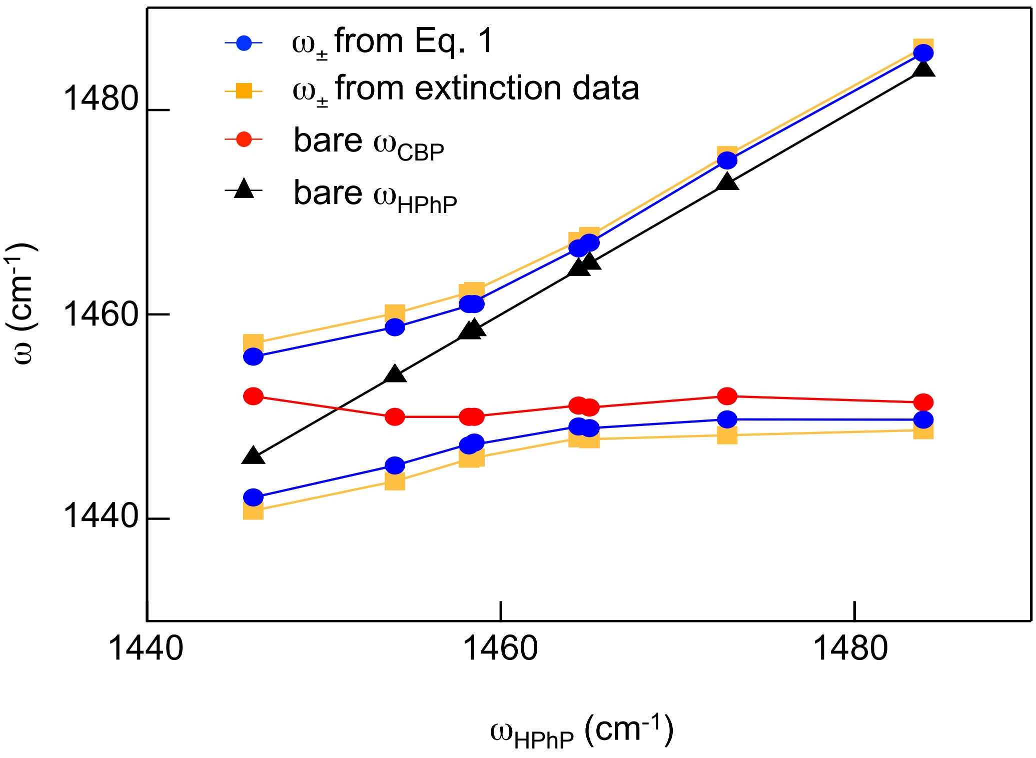

Figure S4. Anti-crossing in the transmission spectra and in the energy spectrum of the coupled modes. Minima of relative transmission dips as extracted from Lorentzian fits (yellow squares). Frequency of upper and lower branches of the mixed state, as extracted from a fit of the coupled oscillators model (blue dots), together with the bare CBP vibrational frequency ω_{CBP} and ω_{HPhP} resonance frequency (red dots and black triangles, respectively). All values are plotted as a function of ω_{HPhP}.

S5 Evaluation of coupling regime of the numerically calculated spectra

Calculated transmission spectra of h-BN ribbons with 30 nm CBP on top were obtained by electromagnetic simulations and are shown as color plot in Figure 4d of the main text. In order to evaluate the coupling regime, we performed the same analysis implemented for the experimental spectra in the main text: we fit single spectra (for several values of ribbon width, *w*) by the coupled oscillators model, extract the coupling strength g and compare it to the losses of the non-interacting modes. Calculated spectra and fits are plotted in Figure S5a, while the extracted bare HPhP and CBP resonance frequencies are shown in Figure S5b, together with the calculated energies for the eigenmodes of the coupled system. All fitting parameters are collected in Table S2. From the fits we are able to extract the coupling strength for each curve. We obtain the average values $\bar{g}$ = 7.4 cm^{-1}, $\bar{\gamma}_{\mathrm{HPhP}}$ = 14.7 cm^{-1} and $\bar{\gamma}_{\mathrm{CBP}}$ = 9.4 cm^{-1}, which yield to C_1 = 1.4 and C_2 = 0.31, reported in the main text.

Sample	ω_{HPhP}	γ_{HPhP}	F_{HPhP}	ω_{CBP}	γ_{CBP}	F_{CBP}	***Offset***	g
**T1**	1438.4	15.4	3.7	1450.4	9.5	-0.07	0.05	6.6
**T2**	1447.8	15.2	3.7	1450.9	9.6	0.1	0.05	7.1
**T3**	1455.1	13.2	3.5	1452	9.8	0.14	0.04	7.8
**T4**	1462.6	13	3.3	1451.6	9.8	0.36	0.04	7.8
**T5**	1468.5	15.3	3.2	1451.7	8.8	0.33	0.03	7.8
**T6**	1474.6	15.8	3.1	1451.2	9	0.46	0.03	7.1

Table S2. Parameters of the coupled oscillators model to fit the simulated transmission spectra. All parameters are expressed in cm^{-1}, except for *Offset* (adimensional) and $F_{\mathrm{HPhP,CBP}}$ which are expressed in cm^{-2}.

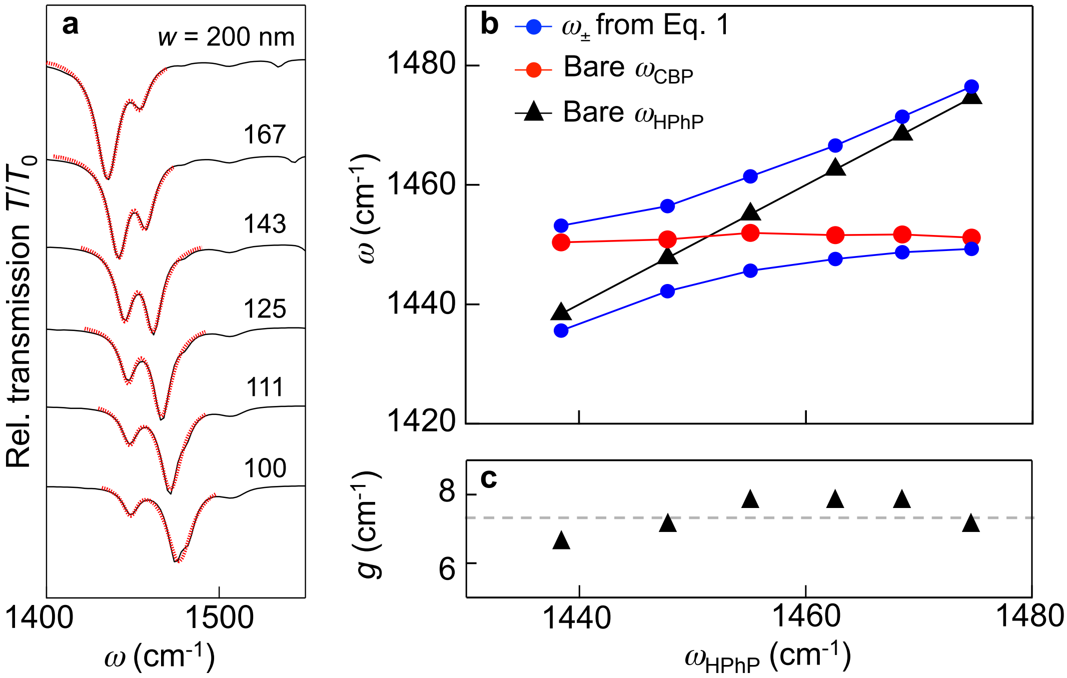

Figure S5. Theoretical spectra: fit to coupled oscillators model. (a) Simulated transmission spectra of 43 nm thick h-BN ribbon arrays with fixed period $D = 400$ nm and variable width w with 30 nm of CBP evaporated on top (black). Red dotted lines are fit of the coupled oscillators model. (b) Bare frequencies of HPhP and CBP resonances (ω_{HPhP} and ω_{CBP}, respectively) as extracted from the fits and eigenmodes frequency calculated according to Equation 1 of the main text, plotted as a function of ω_{HPhP} (c) Values of the coupling strength g, as extracted from the fits.

1 Glover III RE, Tinkham M. Conductivity of superconducting films for photon energies between 0.3 and 40 kT_c. *Phys Rev* 1957; **108**: 243-256.

2 Dressel M, Grüner G. *Electrodynamics of Solids: Optical Properties of Electrons in Matter*. Cambridge University Press, UK, 2002.

3 Glaser T, Beck S, Lunkenheimer B, Donhauser D, Köhn A *et al*. Infrared study of the MoO_3 doping efficiency in 4,4′-bis(N-carbazolyl)-1,1′-biphenyl (CBP). *Org Electron* 2013; **14**: 575-583.
